# RNA-Targeted Therapies and Amyotrophic Lateral Sclerosis

**DOI:** 10.3390/biomedicines6010009

**Published:** 2018-01-15

**Authors:** Stéphane Mathis, Gwendal Le Masson

**Affiliations:** 1 Department of Neurology, Nerve-Muscle Unit and ALS Center, CHU Bordeaux (Pellegrin Hospital), F-33000 Bordeaux, France; gwendal.le-masson@chu-bordeaux.fr; 2 Neurocentre Magendie, Physiopathologie de la Plasticité Neuronale, University of Bordeaux, U862, F-33000 Bordeaux, France; 3 INSERM, Neurocentre Magendie, Physiopathologie de la Plasticité Neuronale, U862, F-33000 Bordeaux, France

**Keywords:** antisense oligonucleotide (ASO), ASOs, therapy, RNA, ALS

## Abstract

Amyotrophic lateral sclerosis (ALS) is a fatal motor disease in adults. Its pathophysiology remains mysterious, but tremendous advances have been made with the discovery of the most frequent mutations of its more common familial form linked to the *C9ORF72* gene. Although most cases are still considered sporadic, these genetic mutations have revealed the role of RNA production, processing and transport in ALS, and may be important players in all ALS forms. There are no disease-modifying treatments for adult human neurodegenerative diseases, including ALS. As in spinal muscular atrophy, RNA-targeted therapies have been proposed as potential strategies for treating this neurodegenerative disorder. Successes achieved in various animal models of ALS have proven that RNA therapies are both safe and effective. With careful consideration of the applicability of such therapies in humans, it is possible to anticipate ongoing in vivo research and clinical trial development of RNA therapies for treating ALS.

## 1. Introduction

Amyotrophic lateral sclerosis (ALS) is a rare neurodegenerative disorder. In Europe, the estimated prevalence of ALS is between 2.6 and 3 cases per 100,000 people [1]. ALS is typically characterized by progressive, usually asymmetric, weakness and atrophy of the skeletal muscles, including limb, bulbar, thoracic, and abdominal muscles; oculomotor muscles and sphincter function are typically spared. These symptoms are associated with corticospinal tract damage (spasticity, brisk reflexes, and extensor plantar response) [1]. ALS is a consequence of degeneration of both central and peripheral motor neurons. Over the past decade, compelling clinical, imaging, and neuropathological data have provided evidence that the disease process involves not only motor neurons but also non-neuronal cells such as astrocytes and microglia [1]. ALS is no longer considered a strictly motor degenerative disorder because non-motor symptoms, including cognitive dysfunction, progressive supranuclear palsy-type eye movements, extrapyramidal features and sensory disturbance with small fiber neuropathy, are now recognized as part of the disease [2,3].

From a management point of view, the emergence of multidisciplinary, specialized ALS clinics has increased both the quality of life and the survival of ALS patients: respiratory management through early non-invasive ventilation is probably one of the most important determinants of survival. However, although many preclinical studies have been conducted in animal models within the last two decades [3,4], none have successfully identified effective therapeutics, except for riluzole which has been shown to extend survival by three months and the time to tracheostomy; but the disease still results in mortality within 2–5 years [5]. Recently, the free radical scavenger edaravone was approved for ALS by the US Food and Drug Administration (FDA) [6], and the tyrosine kinase inhibitor masitinib is another potential treatment candidate for ALS (phase 3 development) [4,7]. However, further research and therapeutics are needed.

Optimization of treatments for ALS could require multiple cell type and pathogenic mechanism targets [3]. As an effective method for gene downregulation that may be applicable in multiple neurodegenerative diseases, ribonucleic acid (RNA)-targeted technology may provide a potential therapeutic strategy for ALS (Table 1).

## 2. Principle of RNA-Targeted Therapies in Amyotrophic Lateral Sclerosis (ALS)

Gene therapy generally refers to the transfer of a therapeutic gene into a target tissue and the maintenance of gene function for a sufficient length of time. The RNA-targeted approach, which includes antisense oligonucleotides (ASOs) and small interfering RNA (siRNA) methods, involves designing short oligonucleotides per Watson-Crick base-pairing rules to target specific mRNAs in a cell. Small interference RNA (siRNA) and small hairpin RNA (shRNA) are exogenous small RNAs, in contrast to endogenous microRNA (miRNA) [25]. The desired outcome of antisense oligonucleotide (ASO) technologies is degradation of the targeted mRNA: the end result is highly specific inhibition of de novo synthesis of a specific protein product. ASOs have also been used in exon skipping techniques to fully block protein translation. The difference between ASO drug therapies and gene therapies is the absence of stable changes in the genome and the consequent requirement for repetitive exogenous administration of the antisense compounds [26]. The main differences between ASOs and siRNA are the enzymes that degrade RNA, their sites of action, and the different methods used to apply ASOs or siRNA therapeutically. The endogenous physiological mechanism of the cell called RNA interference (RNAi) primarily occurs in the cytoplasm via the RNA-induced silencing complex (RISC): siRNA can be continually produced from a viral vector delivered to animal models and potentially to humans; for shRNA, the RNAi cytoplasmic process is the same as that for siRNA, but shRNA is constantly synthesized in host cells with long-term gene silencing [25]. ASOs are short, chemically modified, synthetic nucleic acids that activate ribonuclease H, inhibit 5′ cap formation, arrest mRNA translation in the cytoplasm and alter mRNA splicing; ASOs also block access to pre-mRNA and mRNA and prevent RNA folding, but ASOs do not activate degradation of the target mRNA [27,28,29,30]. Due to recent improvements in ASO structure, we have observed a re-emergence of ASOs as valuable tools that provide information regarding disease mechanisms and as powerful therapeutics for disease intervention. Because ASOs are distributed widely throughout the central nervous system (CNS), e.g., the brain and spinal cord, when delivered to the cerebrospinal fluid (CSF), ASOs currently represent a potential therapeutic strategy for CNS disorders such as Huntington’s disease, primary tauopathies, Alzheimer’s disease, spinal muscular atrophy (SMA) and ALS [31]. However, unlike siRNA, which can be delivered via peripheral injection, ASOs do not cross the blood-brain barrier and must be delivered directly to the CNS via intrathecal injection [11].

Spinal muscular atrophy (SMA) is an inherited motor neuron disorder caused by a reduced level of survival motor neuron (SMN), a crucial protein of spliceosome subunit biogenesis, due to deletions or mutations of the *SMN1* gene. In humans, because of the presence of the duplicate gene *SMN2*, which is similar to *SMN1* but also different from it due to a C to T substitution in exon 7, SMA severity may be modified by the number of copies of *SMN2* [32]. SMA is a recent example of the efficacy of splicing correction therapies in humans. Using ASOs, manipulation of pre-messenger RNA (mRNA) splicing is possible, and the approach enables modification of defective transcripts from the *SMN2* gene to induce gene transcription and translation into the SMN protein and restore SMN function. In December 2016, after successful results were obtained in children with SMA [33], the FDA approved SPINRAZA^TM^ (Nusinersen) as the first and only genetic treatment to date for SMA; this ASO is designed to bind to *SMN2* pre-mRNA and promote the inclusion of exon 7. Consequently, a similar approach has been proposed for familial and sporadic ALS patients in whom a causative gene has been identified [34].

The mechanisms underlying neurodegeneration in ALS are not fully understood. Multiple cellular and molecular processes have been implicated, such as excitotoxicity and oxidative stress, and the role of sequestration of RNA-binding proteins is crucial in ALS [1,3,35]. Most ALS cases are sporadic, probably due in part to multiple genetic and environmental risk factors. Abnormal aggregation of 43-kDa trans-activation response (TAR) DNA-binding protein (TDP-43) is a hallmark pathological feature in nearly all ALS patients, except for patients carrying mutations in the *superoxide dismutase 1* (*SOD1*) and *fused in sarcoma* (*FUS*) genes [1]. Less than 10% of ALS cases are classified as familial ALS (fALS), with more than 30 genes presumably involved in the ALS etiology and pathogenesis [36]. In approximately 60 to 80% of fALS patients, a high-impact, presumably pathogenic mutation can be identified, with *C9ORF72* (40%), *SOD1* (20%), *FUS* (1–5%), and *TARBDP* (1–5%; coding for TDP-43) as the most frequent [1]. Recently, small binding molecules that specifically target *C9ORF72* GGGGCC repeat RNA G-quadruplex have been identified; by targeting this hexanucleotide in a *C9ORF72* model of drosophila, the authors improved the survival of these fruit flies, suggesting that targeting RNA may be a therapeutic option in ALS [37]. Both *C9ORF72* and *SOD1* have been the leading therapeutic targets for ALS and have provoked preclinical research and clinical translation of ASO therapies to human patients [31]. Epigenetics represents potential convergence between genetic predisposition and environmental exposure. Multiple epigenetic mechanisms have been found in ALS, including methylation, hydroxymethylation, histone modification, RNA editing and post-transcriptional silencing mechanisms [38]. The pathophysiology of *C9ORF72* genetic abnormalities clearly emphasizes the role of RNA and protein metabolism disruption, and increasing knowledge regarding the different forms of RNA and their corresponding functions has paved the way for RNA-based medicine which is defined as the therapeutic targeting of RNA. Patients with neurodegenerative diseases such as ALS are already benefiting from these new approaches. Two major applications will most likely be developed: patient profiling using microRNA screening and inhibition of protein synthesis through RNA therapy. ALS has increasingly been considered a proteinopathy with prion-like mechanisms, a concept that is currently applied to many neurodegenerative diseases, such as Parkinson’s and Alzheimer’s diseases, in which protein aggregation is a key feature.

## 3. Biomarkers and microRNA Screening in ALS

Reliable diagnostic biomarkers are needed to reduce diagnostic delays (which are excessively long) to facilitate early initiation of treatment [1]. Clinical tools such as the ALS-Functional Rating Scale-Revised (ALS-FRS-R) [39], which is a common tool for the clinical evaluation of ALS patients, are usually used as primary outcome measures in clinical trials. The Medical Research Council (MRC) is used for muscle strength evaluations, and pulmonary function tests are used to evaluate respiration [1]. Electrophysiological techniques such as motor unit number estimation [40] or the motor unit index [41] may help evaluate muscle innervation. Transcranial magnetic stimulation may be helpful for examining the corticospinal pathway. Diffusion tensor magnetic resonance imaging [42] and fluorine-18-fluorodeoxyglucose positron emission tomography [43] have been proposed as diagnostic biomarkers.

However, despite numerous efforts, the number of reliable biological biomarkers for ALS remains limited. Although the incidence of fALS is close to 10%, detection of mutations is not a reliable diagnostic approach because of the unknown pathogenicity and penetrance of the genes and possible oligogenecity, whereby the interactions of more than one gene determine the phenotype [44]. The neurofilament, which is a marker of axonal degeneration, is one of the most promising biomarkers in ALS. The levels of phosphorylated heavy and light neurofilaments are high in the CSF and serum of ALS patients, and the neurofilament light chain level is a good prognostic marker for ALS, with high levels in the CSF corresponding with short survival [22]. The serum creatine kinase level may be an independent prognostic factor for survival in ALS [45]. Finally, because abnormal aggregation of TDP-43 is a pathological hallmark of the disease, determining specific TDP-43 variants to provide additional ALS biomarkers has recently been proposed [46].

MicroRNA (miRNA) is a tissue-specific, small RNA that regulates the translation of protein-coding RNA, accounting for 1 to 2% of non-protein-coding genes [47,48]. Dysregulation of miRNA-related pathways in the CNS leads to severe neuronal injury and cell death: their roles are crucial in many neurodegenerative disorders due to their involvement in developmental, functional and survival functions of various mature neuron types [48]. Several findings suggest that ALS patients show dysregulated gene expression profiles. Dysregulated miRNA and circulating miRNA profiles have been identified with homogeneous signatures in the sera of fALS patients and in pre-symptomatic carriers of *ALS* gene mutations. Conversely, miRNA signatures of sporadic ALS are highly heterogeneous, suggesting multiple etiologies. Therefore, sporadic ALS may involve a higher contribution of genetic factors than expected [49]. A small regulatory miRNA (miR-218) has recently been identified as highly enriched in and exclusive to motor neurons; as miR-218 is detectable in the spinal tap biofluid of an ALS rat model, with the levels changing as the disease progressed, miR-218 may be a clinically useful marker of disease status [50]. Another miRNA involved in the maintenance of neuromuscular connectivity in ALS (miR-206) is elevated in the circulation of symptomatic *SOD1*^G93A^ mice and possibly in human ALS patients [51]. Indeed, miR-155, which plays an important role in inflammatory responses, is overexpressed in the *SOD1* mouse and in ALS and fALS patients. Moreover, restoration of dysfunctional glia and improvements in motor symptoms have been observed when miR-155 was targeted in *SOD1* mice [18]. Finally, in other diseases (e.g., cancer and hepatitis) [52], inflammatory-related miRNAs, such as miR-155, and other markers of inflammation (e.g., connexin-43 and pannexin-1) represent interesting pharmacological targets for the characterization of neuroprotective treatments in ALS patients [53]. The use of miRNAs as disease biomarkers may have strong potential not only for early diagnosis but also for predicting disease severity and evolution and for improving phenotype classification and group stratification in clinical trials.

## 4. RNA-Targeted Therapy in ALS

### 4.1. RNA-Targeted Therapy and SOD1-Related ALS

Although dominant mutations in the *SOD1* gene are causes of fALS, *SOD1* was the first gene identified as associated with this disease; more than 170 *SOD1* missense mutations have been identified since 1993 [54]. The SOD1 protein is an antioxidant found in the cytosol and mitochondrial intermembrane space, and *SOD1* converts superoxide radicals to hydrogen peroxide. In vitro and in vivo models based on *SOD1* mutations are still widely used to investigate ALS mechanisms [3]. However, the effects of the mutations on *SOD1* enzymatic activity do not correlate with disease characteristics. Moreover, mouse data demonstrate that mutant *SOD1* expression causes motor neuron disease, whereas genetic deletion of *SOD1* does not. These data indicate that the toxicity of *SOD1* is the result of a gain in toxic function associated with the aggregation of misfolded *SOD1* rather than a loss of enzymatic function [55]. Reducing the concentration of the mutant protein is predicted to slow the progression of *SOD1*-related ALS [56]. However, in *SOD1* mutant mice, toxicity to motor neurons is non-cell autonomous; specifically, wild-type non-neuronal cells are able to delay or eliminate the degeneration and death of mutant-expressing motor neurons, while wild-type neurons acquire damage from mutant-expressing neighbors [57].

By injecting siRNA intramuscularly for delivery to spinal motor neurons through retrograde transport of adeno-associated virus (AAV), one study demonstrated a substantial decrease in *SOD1* and a subsequent functional impact, namely a delayed loss of grip strength, in the *SOD1*^G93A^ mouse model of ALS, providing proof of principle for the selective reduction of any neuronal protein and supporting intramuscular injections of siRNA for fALS [9]. Indeed, intraspinal or intramuscular injections of lentiviruses with shRNA *SOD1* increase retrograde transport, delay the onset of motor neuron symptoms (115%) and extend survival (77% of normal life) in *SOD1*^G93A^ mice [10]. This result has been confirmed by intrathecal and peripheral injections of an AAV encoding shRNA against human *SOD1* mRNA, which delayed disease onset and progression in *SOD1*^G93A^ mice (by approximately 30–40%) and nonhuman primates [13,19,58]. Recently, the combined use of intravenous and intracerebroventricular delivery of AAV10-U7-h*SOD1* (an AAV serotype rh10 used to mediate exon skipping of the human *SOD1* pre-mRNA by expression of exon-2-targeted antisense sequences embedded in a modified U7 small-nuclear RNA) produced an increase in the survival rate of *SOD1*^G93A^ mice injected at birth (92%) or at 50 days of age (58%) and prevented weight loss and neuromuscular functional decline [22].

IONIS Pharmaceuticals (“ISIS Pharmaceuticals”until December 2015) proposed the use of ASOs (ISIS333611) as a therapeutic strategy for ALS patients carrying mutations in the *SOD1* gene. A study of an ALS *SOD1*^G93A^ animal model showed the following: (a) widespread distribution of intrathecally delivered ISIS333611; (b) reduced *SOD1* mRNA and protein levels in the CNS; and (c) increased survival of the mutant mice [11]. Recently, intrathecal single-dose ISIS333611 infusion was administered to four cohorts of eight patients with *SOD1*-related ALS: in each cohort, six patients received ISIS333611 and two patients received a placebo [16]. Although conclusions were limited due to the small doses and small number of patients studied, the main results of this study were as follows: (a) ISIS333611 has no dose-limiting toxic effects or other safety and tolerability concerns; and (b) the CSF and plasma drug concentrations were consistent with those predicted in preclinical studies [16]. These results confirm that ASOs delivered to the CNS represent a feasible treatment for *SOD1*-related ALS and that ASOs can be used in cases of sporadic ALS to compensate for the amount of misfolded *SOD1*.

Clustered Regularly Interspaced Short Palindromic Repeats (CRISPR) and the associated Cas9 protein, encoded by archaea and bacteria, are vital components of the adaptive immune system. The discovery of the CRISPR-Cas system in 1987 [59] resulted in gene therapy applications and the adaptation of the type II CRISPR system from *Streptococcus pyogenes* for genome editing, particularly since 2012 [60]. Despite the imperfection of this genome editing approach, completely depending of RNA-guided endonucleases and protospacer adjacent motif (e.g., the DNA sequence immediately following the DNA sequence targeted by the Cas9 nuclease), it represents a potential powerful medical tool [61]. Recently, using the CRISPR/Cas9 system, Wang et al. targeted genes to introduce correction mutations in induced pluripotent stem cells from fibroblasts of fALS *SOD1* and *FUS* patients; their results were confirmed by genomic DNA sequencing for *SOD1* [23].

### 4.2. RNA-Targeted Therapy and C9ORF72-Related ALS

Hexanucleotide (GGGGCC) repeat expansions in the *C9ORF72* gene have emerged as an important cause of ALS, frontotemporal dementia (FTD) and the combination of ALS and FTD (ALS-FTD), but such expansions are also found in a significant minority of patients with sporadic ALS [1,35]. The exact mechanism by which abnormal GGGGCC expansion mediates disease pathogenesis remains unknown. The loss of normal *C9ORF72* function and gains of RNA toxicity and protein toxicity have been suggested [62]. Testing of potential therapeutics, especially ASO therapy, was initially complicated by the lack of a *C9ORF72* rodent model. With the availability of human-induced pluripotent stem cell-derived neurons and fibroblasts from *C9ORF72*-related ALS patients, followed by the recent development of a mouse model expressing the expanded *C9ORF72* gene, the study of ASOs in *C9ORF72*-related ALS has become possible [31].

ASOs, which bind within the repeat GGGGCC expansion or within the surrounding *N*-terminal regions of the *C9ORF72* mRNA transcript, fail to reduce RNA levels; however, ASOs effectively reduce toxic RNA foci, restore normal gene expression markers and protect against glutamate toxicity [12,15,17]. Moreover, studies in animal models of *C9ORF72*-related ALS have shown that RNA and protein toxicity are crucial elements in the pathophysiology of ALS, strengthening the possibility of the therapeutic use of ASOs in ALS [31]. However, the exact function of the 54-kDa *C9ORF72* protein is largely unknown, so the safety of blocking protein expression with ASO-based treatments must be carefully evaluated. A chronic, partial reduction in *C9ORF72* does not provoke ALS, but the absence of *C9ORF72* induces splenomegaly, enlarged lymph nodes and mild social interaction deficits, but no motor dysfunction. Conversely, GGGGCC expansions cause accumulation of RNA foci and dipeptide-repeat (DPR) proteins, loss of hippocampal neurons, increased anxiety, and impaired cognitive function [20]. The production of aberrant DPR proteins via repeat-associated non-AUG-dependent translation is a proposed disease mechanism for *C9ORF72* repeat expansions [63]. Decreased protein levels, obtained via knockdown of the *C9ORF72* ortholog, cause motor defects in zebrafish, confirming that *C9ORF72* is essential for proper development and motor system function [64]. An aberrant splicing mechanism is presumed to underlie *C9ORF72* pathogenesis, even when splicing alterations have not been clearly recognized in tissues or cells carrying mutated *C9ORF72* [12]. A recent study has shown that both the sense and antisense strands of *C9ORF72* are transcribed in fibroblasts from patients with the repeat expansions, suggesting that ASOs may need to target both sense and antisense RNA foci to be therapeutically effective [15]. Finally, the use of a single dose of ASOs to target repeat-containing RNA while preserving mRNA levels and encoding *C9ORF72* in mice provokes (a) significant accumulation of sense RNA foci (without increasing the antisense foci) and (b) significant amelioration of behavioral deficits (even 6 months after the single injection) [20].

### 4.3. RNA-Targeted Therapy and the ATXN2 and TARDBP Genes

Spinocerebellar ataxia type 2 (SCA2) is an inherited disorder caused by polyglutamine (polyQ) tract expansions secondary to mutations of the *ATXN2* gene (coding for the Ataxin-2 protein); these polyQ expansions are encoded at the DNA level in *ATXN2* by trinucleotide CAG repeats [65]. The RNA-binding protein Ataxin-2 has multiple roles in RNA metabolism, especially in the regulation of stress granule assembly, and the concentration of aggregation-prone proteins, such as TDP-43, in stress granules may lead to the formation of pathological protein aggregates in ALS. TDP-43 is the major disease-associated protein of ALS and is present in the ubiquitinated cytoplasmic inclusions of the neurons of 97% of ALS patients, 50% of whom have frontotemporal lobar degeneration (FTD) [62]. Subsequently, mutations in the *TARDBP* gene (encoding TDP-43) are associated with some sporadic and familial cases of ALS and FTD with ubiquitinated inclusions, indicating a central role of TDP-43 in ALS pathogenesis [66,67]. In yeast and flies, a decrease in Ataxin-2 suppresses TDP-43 toxicity, but CAG expansions in *ATXN2* increase the risk of ALS [21]. Therefore, *ATXN2* plays an important role in the predisposition to ALS, and polyQ expansions in Ataxin-2 are a significant risk factor for the disease [68,69].

The administration of ASO-targeting *ATXN2* to the CNS of *TARDBP* transgenic mice markedly extends their lifespan and improves their motor performance [21]. The approach, which involves targeting a modifier gene that does not directly cause ALS and reducing Ataxin-2 levels, represents an original therapy for TDP-43 proteinopathy: this type of approach will likely be essential for treating sporadic ALS because of the cellular role of TDP-43, which is present in nearly all ALS patients.

## 5. Limitations of RNAi-Based Therapeutic Strategies

As agents that focus on specific cells, RNAi-based technologies are promising therapeutics for various diseases, such as neurodegenerative disorders and cancer. However, due to the pharmacodynamic properties of these technologies, other consequences of the systemic administration of siRNA include the induction of numerous interactions throughout the body, leading to potential side effects and making the safe and effective delivery of such substances questionable [70]. Because siRNAs may be recognized by the immune system, with complex interactions via pathogen recognition receptors, the siRNA oligonucleotides and their delivery vehicles (especially lipoplexes and polyplexes) can be immunostimulatory [71]. Moreover, the saturability of the RNAi pathway has been demonstrated in several studies [72,73,74,75,76] in which potential toxic effects could be avoided by limiting the dose of exogenous RNA [77]. Finally, the lack of specificity and the potential toxicity of RNAi-based technologies represent serious obstacles to the therapeutic use of such substances in routine care.

However, the major difficulty in neurodegenerative disorders is that ASOs do not cross the blood-brain barrier. Consequently, the intrathecal route of administration, which is invasive by nature, leads to the risk of adverse effects such as post-lumbar puncture headache or post-surgical complications. Considering the advances in the field of the nanotechnologies, certain solutions have been proposed to facilitate the distribution of ASOs through the CNS. Recently, to improve the successful delivery of *SOD1* ASOs to motor neurons of zebrafish, some authors have proposed the use of calcium phosphate-lipid nanoparticles because of their capacity to encapsulate ASOs directed to *SOD1* [24].

## 6. Conclusions

The application of RNA-based drugs to modulate gene/protein expression represents an important goal for ASO technology in the treatment of neurodegenerative disease. After more than two decades of development, RNA therapeutics have become a clinical reality and may serve as potential strategies for various diseases, including neurodegenerative disorders: recently, an effective RNA-targeted therapy has been proposed for SMA. The application of RNA-based drugs for the modulation of gene/protein expression represents an important benchmark for ASO technology in terms of therapy for neurodegenerative disease. At present, no RNA therapeutics have been found to be effective for ALS, but such a therapeutic approach could be considered in the future for the treatment of both familial and sporadic ALS.

## Figures and Tables

**Table 1:** Main RNA-therapy studies in amyotrophic lateral sclerosis.

Year	Reference	Organism			Gene Target				Therapeutic Strategy		
		Animal	Human Cells	Human	*SOD1*	*C9ORF72*	*FUS*	*ATXN2*	*ASOs*	*SMT*	*GT*
2003	Ding et al. [8]	D			+					+	
2005	Miller et al. [9]	M			+					+	
2005	Ralph et al. [10]	M			+					+	
2006	Smith et al. [11]	R/NHP			+				+		
2013	Donnelly et al. [12]		+			+			+		
2013	Foust et al. [13]	M			+					+	
2013	Koval et al. [14]	M			+					+	
2013	Lagier-Tourenne et al. [15]	M	+			+			+		
2013	Miller et al. [16]			+	+				+		
2013	Sareen et al. [17]		+			+			+		
2015	Butovsky et al. [18]	M			+					+	
2016	Borel et al. [19]	M/NHP			+					+	
2016	Jiang et al. [20]	M				+			+		
2017	Becker et al. [21]	M						+	+		
2017	Biferi et al. [22]	M			+					+	
2017	Wang et al. [23]		+		+		+				
2017	Chen et al. [24]	Z			+				+		

ASOs: antisense oligonucleotides; D: drosophila; GT: gene therapy; M: mouse; NHP: nonhuman primate; R: rat; SMT: small molecule therapy; Z: zebrafish.
